# Spanish Society of Medical Oncology recommendations for comprehensive assessment and care of cancer survivors’ needs

**DOI:** 10.1007/s12094-024-03571-9

**Published:** 2024-07-08

**Authors:** Alejandro Gallego, Carmen Beato, Elena Brozos, Susana De La Cruz, Ruth Vera García

**Affiliations:** 1 Department of Medical Oncology, Cancer Center Clínica Universidad de Navarra (CCUN), Madrid and Pamplona, Calle del Marquesado de Santa Marta, 1, 28027 Madrid, Spain; 2 Departament of Oncology, University Hospital of Jerez de La Frontera, Cádiz, Spain; 3 Department of Oncology, University Hospital of A Coruña, A Coruña, Spain; 4 Department of Oncology, University Hospital of Navarra, Instituto de Investigación Sanitaria de Navarra, IdISNA, Pamplona, Spain

**Keywords:** Cancer survivor, Care models, Future strategies, Survivor needs

## Abstract

This article reviews the contemporary and inclusive definition of cancer survivorship, including patients with and without disease who have completed or continue to undergo treatment. The Spanish Society of Medical Oncology (SEOM) describes in this article the needs of these patients and outlines a care model based on an estimation of cancer incidence and identification of patient needs, to enable the provision of practical actions to achieve effective care. The objectives of this review are to identify the main effects of cancer on survivors and to establish appropriate ways of measuring these effects, as well as discussing the management of physical, psychological and social, occupational, financial, and other health-related needs. We suggest a multidisciplinary care model and training programs for the different professionals involved in care, and highlight challenges and the future role of the SEOM and health-care policy in ensuring optimum care of cancer survivors.

## Introduction

Owing to advances in cancer screening of at-risk individuals, diagnosis, treatment, and supportive care, the mortality associated with cancer is decreasing and the number of cancer survivors, i.e., people living after a cancer diagnosis, is growing around the world [1]. Moreover, based on 2020 estimates and global demographic projections, the incidence of cancer is expected to rise by 47% between 2020 and 2040, bringing the global cancer burden to a total of 28.4 million cases worldwide by 2040 [2].

The prevalence of “cancer survivors” is difficult to quantify due to differences in survivorship definitions and data availability across countries; however, data from the EUROCARE-6 study suggest that there were 22.7 million people in Europe living with a history of cancer in 2020 (a 46% increase from the 15.5 million people estimated in 2010) [3]. Moreover, the number of people in Europe living > 10 years after a cancer diagnosis increased over the last decade, from 4.9 million (31%) in 2010 to 8.2 million (36%) in 2020 [3]. While the epidemiology of cancer survivorship in Spain is not well described, the Global Cancer Observatory estimates that there were over 282,000 new cases of cancer diagnosed in Spain in 2020 (out of 19.3 million globally), and more than 858,000 people had been diagnosed in the previous 5 years (out of 50.6 million globally) [2, 4, 5]. Also, the total prevalence of individuals with cancer in Spain was estimated at 2,265,152 (including 1,066,959 males and 1,198,193 females) in 2020 [6].

As the number of people living with and beyond diagnosis of cancer continues to grow, the healthcare system burden also increases. Cancer survivors face a wide range of physical, psychological, social, occupational, and financial challenges that persist long after diagnosis and treatment, and cannot be fully addressed in the everyday clinical practice with current assisting models [7–9]. With that in mind, in 2013 the Spanish Society of Medical Oncology (SEOM) developed a comprehensive plan to improve cancer survivorship care in Spain, by promoting research, professional training, patient education, and multidisciplinary care for persons who are alive and free of disease 5 years after diagnosis and treatment [10]. However, there is increasing recognition that the concept of survivorship, and the evaluation of adverse effects and the needs of care that accompanies this, must be addressed from the time of diagnosis [11–15].

Herein, we present updated SEOM recommendations for cancer survivorship, including a revised definition of “cancer survivor”, a review of the challenges and unique care needs of this population, and guidance to improve the ongoing health and wellbeing of people living after a cancer diagnosis.

## Cancer survivorship definition

In 1986, the US National Coalition for Cancer Survivorship first used *cancer survivor* to describe an individual “from the time of diagnosis and for the balance of life” [12, 13]. This concept of lifelong survivorship has since been widely adopted, including in the US NCCN Clinical Practice Guidelines in Oncology (NCCN Guidelines®), which define *cancer survivor* as any person with a history of cancer, including “those who are initiating treatment, in ongoing treatment, have completed cancer treatment, or are in clinical remission” [15]. Similarly, the American Cancer Society uses *cancer survivor* to describe anyone with a past or present cancer diagnosis, regardless of where they are in the course of their disease [11], and the European Society for Medical Oncology (ESMO) agrees that cancer survivorship starts at the date of diagnosis, irrespective of treatment intent [14]. Because SEOM acknowledges that, in Spain, the term “cancer survivor” described in the 2013 national guidelines could be misleading or even uncomfortable for some patients, current guidelines adopt the broader concept and align with the consensus in Europe and the United States (US) [11, 14, 15] and our own definition of surviving, i.e., living after a certain event (in this case, the diagnosis of cancer). Although this definition of survivorship is intended to capture a broad population of those living with, through, and beyond cancer, it may not always be useful from a practical perspective, because the heterogeneity of the overall population of cancer survivors results in wide-ranging and evolving care needs. Therefore, it may be more beneficial to define and identify different types of cancer survivors, so that tailored management strategies can be developed and implemented for each group dependent on the phase of survivorship.

**Fig. 1:**
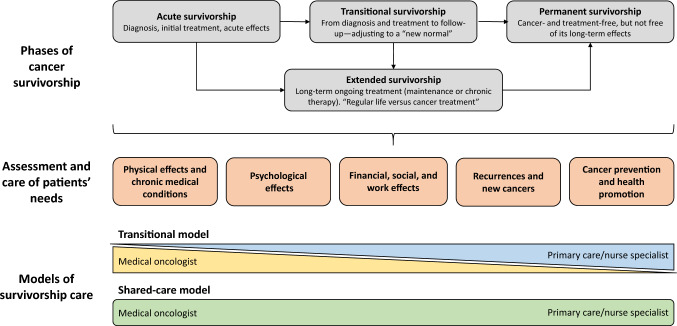
Phases, needs, and models of care for cancer survivorship

The phases of survivorship first described in the 1980s have since been revised and expanded to reflect improvements in cancer screening, diagnosis, treatment, and prognosis in recent decades (Fig. 1) [16]. After the acute phase of diagnosis and initial treatment, patients immediately enter a new phase of “transitional survivorship”, which may include a period of watchful waiting in those who respond to initial treatment, or a period of readjustment for those not in remission or with stable or progressive disease. The third phase of “extended survivorship” includes patients in remission not receiving maintenance therapy, those who are cancer-free due to ongoing treatment, and those living with advanced cancer as a chronic disease. It is the recommendation of the Authors that patients receiving maintenance therapy also be included in this group as 3–5 years of maintenance therapy seems too long to consider them as being in the transitional phase. The fourth phase of “permanent survivorship” encompasses patients who are cancer-free, but who may not be free of the long term effects of cancer and its treatment. Since the needs of patients in each of these phases differs, in this updated SEOM guidance document, we provide recommendations for the optimal care of people in the transitional, extended, and permanent phases of survivorship. Acute effects should be managed according to cancer-specific clinical guidelines.

## Cancer survivors’ needs

As stated before, people with cancer may experience a range of secondary effects while living through and beyond their diagnosis, which can negatively impact their physical, psychological, social, occupational and financial functioning, and overall quality of life (QoL) [1, 17]. While some effects may be temporally associated with the initial diagnosis and treatment of cancer, others may develop during and persist after treatment, or emerge months or years after treatment has ended. As mentioned previously, this article discusses the care needs of survivors who have developed effects after initial treatment completion (Table 1). These care needs are individual to each patient, depend on the pathology and type of treatment received (e.g., surgery, chemotherapy, radiotherapy), and may evolve or change in intensity as cancer survivors move through the transitional, extended, and permanent phases of survivorship (Fig. 1). It is also possible that adverse effects of treatment can trigger other side effects (e.g., sexual dysfunction as a physical alteration may trigger psychological or social problems).

**Table 1:** Common needs and adverse events in cancer survivors: how to assess them and their management and care

Most frequent needs/adverse effects	Assessment of patients’ needs	Management and care
Pain	Numerical rating scale [89] Faces pain rating scale [89] PAIN-B [89] Impact of pain [89]	Assess at each follow-up visit [89] In the event of new or acute pain, assess for cancer recurrence or therapy late effects [15, 22] Pharmacological pain relief (non-opioid adjuvant analgesics, opioids) [15, 17, 22] Non-pharmacological strategies (exercise, acupuncture, heat and cold, massage, physiotherapy) [15, 17, 22, 89]
Peripheral neuropathy	WHO Common Toxicity Criteria for Peripheral Neuropathy [90] NCI Common Toxicity Criteria [90] ECOG Grading Scale for CIPN [90] Quantitative sensory testing [90]	Duloxetine (if chronic and painful) [17] Non-pharmacological strategies (exercise, acupuncture) [17]
Sexual dysfunction	Self-report [23] Female Sexual Function Index [91] DSM-5 [92]	Non-pharmacological (pelvic floor physical therapy, cognitive behavioral therapy, psychosocial counselling, couples therapy) [17] Pharmacological therapy (phosphodiesterase-5 inhibitor, vaginal estrogen) [17] Mechanical therapies [17]
Infertility	–	Individualized pre-treatment oncofertility counselling [28] Referral to fertility specialist or fertility unit for fertility preservation (cryopreservation, gonadal shielding, medical gonadoprotection) [28] Assisted reproductive technology [28]
Osteoporosis	Central/axial DXA [93, 94] Calcaneal DXA [93] Quantitative ultrasound [93] Quantitative CT [30] WHO FRAX [93] Trabecular bone score [30]	BMD scanning (every 2 years) and fracture risk assessment [30, 93, 94] Diet with adequate calcium and vitamin D [17, 30, 93] Lifestyle factors (exercise, smoking cessation, limit alcohol consumption) [17, 30, 93] Bone-modifying agents (bisphosphonates, denosumab) [17, 93] Hormone replacement therapies [30]
Thrombosis	–	Risk factor analysis [33]
Sleep disorders (e.g., insomnia)	WHO ICD-11 [34] APA DSM-5-TR [34] AASM ICSD 3rd edition [34] Insomnia Severity Index [34] Sleep Condition Indicator [34] Pittsburgh Sleep Quality Index [34] Sleep diary [34]	Regular screening using validated tools [34] Sleep hygiene [17] Cognitive behavioral therapy [17, 34] Pharmacotherapy (hypnotics, melatonin) [34] Exercise [34] Bright light therapy [34]
Fatigue	Fatigue Scale-Child [95] Fatigue Scale-Adolescent [95] PedsQL Multidimensional Fatigue Scale [95] PROMIS [95, 96] Brief Fatigue Inventory [96] Fatigue Symptom Inventory [96] EORTC QLQFA13 [96]	Testing with validated fatigue measures [95] Screening for underlying medical conditions (e.g., depression, anxiety, nutritional deficits, pain) [95, 96] Exercise [17] Psychoeducation, mindfulness, cognitive or behavioral therapy [17]
Metabolic syndrome	–	Preventive measures (e.g., dietary counseling, weight loss program, tailored exercise) initiated early in the course of survivorship [17, 74] Referral to specialists [74] Medical intervention to manage underlying pathology (e.g., growth hormone deficiency) or to mitigate risk factors of CVD [17, 74]
Cognitive impairment	Parent-, self-, and teacher-reported measures [97] CogState computerized assessment [97] BRIEF [97]	Neuropsychological assessment [97] Cognitive training and rehabilitation [17, 98] Exercise [17, 98] Mind–body interventions [17] Pharmacotherapy (methylphenidate, modafinil) [98]
Cardiac dysfunction	ECG at 6, 12 and 24 months post-treatment, then if signs or symptoms of cardiac dysfunction are present [17, 99] Cardiac MRI or MUGA scan [100]	Regular review and optimization of CV risk factors [17, 100] Cardiology referral [17] Pharmacotherapies (e.g., ACE inhibitor, ARB, β-blocker) [17, 99] Lifestyle factors (diet, exercise, weight loss) [99]
Lymphedema	Self-report Physical assessment	Manual lymphatic drainage, compression, exercises [17]
Psychological		
Depression and anxiety	NCCN stress thermometer and problem list [15] PHQ-9 [15] GAD-7 [15]	Regular screening for anxiety, depression, trauma, and distress [15] Referral to mental health specialist [15] Non-pharmacological interventions (cognitive behavioral therapy) [15] Pharmacological treatments (antidepressants, anxiolytics) [15]
Fear of recurrence	Self-report	Early recognition, support, and validation of feelings [40] Referral to psychosocial specialist [40] Cognitive behavioral therapy [17, 40]
Post-traumatic stress disorder	Evaluation of patients’ psychiatric and trauma histories at initial clinic visits [41] DSM-5 Trauma- and Stressor-Related Disorders [41]	Psychosocial assessment and support [41]
Social, employment, and financial		
Employment issues	Self-report	“Right to be forgotten” legislation [68, 69] Tailored multidisciplinary intervention (physical, psycho-educational, or vocational) [17]
Financial burden	Self-report	Full early financial disclosure and screening [17] Referral to support services [17] “Right to be forgotten” legislation [68, 69]
Leisure activities (e.g., sports)	Self-report	Tailored exercise program [55]
Relationships and family role	Self-report	Integration of family support into post-treatment supportive care [47] Connecting cancer survivors requiring help to relevant services
Surveillance for recurrence and new cancers		
Surveillance and screening	Individualized genetic testing [14] Tailored screening tests [14]	Testing for hereditary and predisposing syndromes [14] Optimized screening strategies based on individualized benefit–risk profile [14] Increased screening strategies in patients at increased risk of cancer Reduced screening in elderly
Cancer prevention and promotion of overall health and well-being		
Obesity	Physical examination (weight, BMI)	Monitor and promote weight management, healthy diet, and exercise [65, 74] Offer weight loss program and dietary counselling [55, 74]
Smoking	Self-report	Support smoking cessation [65]
Alcohol intake	Self-report	Advise limited alcohol consumption [65]
Vaccinations	–	Vaccination programs in children and adolescents (hepatitis B, HPV) [14, 65]

*AASM* American Academy of Sleep Medicine, *ACE* angiotensin-converting enzyme, *APA* American Psychiatric Association, *ARB* angiotensin receptor blocker, *BMD* bone mineral density, *BMI* body mass index, *BRIEF* Behavior Rating Inventory of Executive Function, *CIPN* chemotherapy-induced peripheral neuropathy, *CT* computed tomography, *CV* cardiovascular, *CVD* cardiovascular disease, *DSM-5* Diagnostic and Statistical Manual of Mental Disorders, fifth edition, *DSM-5-TR* Diagnostic and Statistical Manual of Mental Disorders, fifth edition, text revision, *DXA* dual-energy X-ray absorptiometry, *ECG* electrocardiogram, *ECOG* Eastern Cooperative Oncology Group, *EORTC QLQ-FA13* European Organisation for the Research and Treatment of Cancer Quality of Life Questionnaire-Fatigue13, *FRAX* Fracture Risk Assessment Tool, *GAD-7* General Anxiety Disorder-7 anxiety scale, *HPV* human papilloma virus, *ICD-11* International Classification of Diseases, 11th edition, *ICSD* International Classification of Sleep Disorders, *MRI* magnetic resonance imaging, *MUGA* multigated acquisition, *NCCN* National Comprehensive Cancer Network, *NCI* National Cancer Institute, *PedsQL* Pediatric Quality of Life Inventory, *PHQ-9* Patient Health Questionnaire, *PROMIS* Pediatric Patient-Reported Outcomes Measurement Information System, *WHO* World Health Organization

Qualitative studies have shown that the majority of cancer survivors have approximately 5 unmet needs during the first year after treatment, with almost one-third of patients still experiencing ≥ 1 unmet need 5 years after diagnosis [7, 14]. Specifically, ESMO identified 5 key areas of need which should be systematically addressed in any patient diagnosed with cancer [14]. Each of these 5 key areas is discussed in more detail below.

### Physical effects of cancer and chronic medical conditions

Survivors often need care to manage the physical effects of cancer, whether they are symptoms of the cancer itself or adverse effects of treatment. Chronic pain is one of the most common and debilitating symptoms reported by cancer survivors [18, 19], and may be associated with one or more cancer- or treatment-related causes (e.g., tissue damage, nerve entrapment by tumors, chemotherapy-induced peripheral neuropathy, postoperative pain, sensitization). Neuropathy, a common toxicity associated with a number of cytotoxic drugs (e.g., platinum agents, taxanes, vinca alkaloids, eribulin, among others), is a serious clinical problem which can reduce patient QoL [20, 21]. Other chronic pain syndromes associated with cancer treatment include arthralgias, dyspareunia, and gynecomastia associated with hormonal therapy; chest wall syndrome, cystitis, and plexopathies associated with radiation therapy; and postamputation phantom pain, postmastectomy pain, and pelvic floor pain associated with surgery [1, 22].

Another common physical effect is sexual dysfunction, related to both the physical and psychological impacts of cancer and treatment. Recent systematic reviews and meta-analyses have estimated that the overall prevalence of erectile dysfunction among male cancer survivors is 41% [23], and sexual dysfunction among female survivors is 66% [24]. Also, fertility may be further impaired by gonadal dysfunction caused by specific malignancies (e.g., testicular cancer) or treatment modalities (e.g., cytotoxic chemotherapy, endocrine therapies, pelvic radiation, surgery involving reproductive organs) [25–27]. All patients of reproductive age diagnosed with cancer should receive oncofertility counselling as early as possible [28], at diagnosis or prior to treatment initiation [25, 29]. Counselling should include discussion of methods of fertility/gonadal preservation [25, 28]. Gonadal dysfunction and hormonal disturbances caused by cancer and treatment also play a key role in bone loss and osteoporosis commonly reported among cancer survivors [25, 30]. In addition to osteoporosis [31], other symptoms that may occur after surgical menopause are hot flashes, mood alterations, depression, and insomnia, as well as an impact on sex life, which may cause vaginal dryness, dyspareunia and decreased libido, among others [32]. In addition, we must take into account other adverse effects that may appear in the long term, such as an increase in cardiovascular risk and a greater loss of bone mass, which could lead to osteoporosis.

It is well established that cancer patients are at increased risk of thrombosis [33]. Indeed, improved cancer survival has resulted in an increased incidence of venous thromboembolism (VTE) over the last 20 years [33]. Currently, cancer patients are estimated to have a 12-fold higher risk of VTE than the general population, increasing to 23-fold higher risk in those receiving chemotherapy or targeted therapies [33].

Other physiological effects and care needs associated with cancer survivorship include sleep disorders, fatigue, metabolic syndrome, cognitive impairment, cardiac dysfunction, and lymphedema (Table 1) [1, 17]. Insomnia, a sleep–wake disorder, is the most frequent and clinically significant problem in cancer survivors, being prevalent in up to 30% of patients [34]. Chronic insomnia in cancer survivors is associated with depression, fatigue, pain, and work-related worries [34].

Consideration must also be given to other comorbidities (e.g., diabetes, hypertension) present at diagnosis, which may be exacerbated after cancer treatment. According to a study in more than 300 cancer survivors with a mean age at diagnosis of 56.8 years, 85.9% of patients had at least one associated chronic disease [35].

### Psychological effects of cancer

People living with cancer often require care to address the lasting psychological impact that the diagnosis and treatment of cancer, and its physical effects, can have on them (Table 1). Systematic reviews and meta-analyses have estimated that approximately 12–21% of cancer survivors suffer from depression and 18–21% suffer from anxiety [36, 37]. Moreover, temporary and permanent body changes caused by cancer and its treatment (e.g., hair loss, weight fluctuations, scarring, amputations, ostomies) may negatively impact body image and emotional wellbeing [38].

Depression and anxiety may in part be related to fear of recurrence, which is a commonly reported area of unmet need among cancer survivors [39]. Fear of recurrence or progression is a multidimensional disorder characterized by high levels of preoccupation, and high levels of worry, persistence, and hypervigilance to bodily symptoms [40]. It has been associated with impaired QoL and psychosocial adjustment, elevated emotional distress, and various physical symptoms [40]. Also, post-traumatic stress disorder has been reported in 7.3–13.8% of adult cancer survivors and 12.5% of childhood cancer survivors [41].

### Social, employment, and financial effects of cancer

Cancer survivors often have social and occupational needs due to the physical, psychological, and economic burden of managing cancer and its sequelae. Cancer survivorship has been associated with employment issues such as not returning to work, reduced work hours, and early retirement, in addition to long-term financial hardship [42–45].

Social relationships play a vital role for cancer survivors, strengthening physical functioning through access to information and resources, shared decision-making, and emotional support, and aiding psychosocial well-being and functional independence [46]. In particular, support of family members has been shown to help reduce psychological and emotional distress, improve identification of cancer treatment risks, and increase autonomy and psychological development in younger cancer survivors [47]. However, it has been suggested that some cancer survivors, especially the elderly, may have reduced contact with their social network, resulting in a weakening of ties over time and/or reduced network support which may negatively impact physical functioning [46]. Changed perspectives after their life-altering cancer experiences can also lead to changed priorities that can affect family or social roles [48].

### Surveillance for recurrence and new cancers

Cancer survivors are at risk of recurrence as well as having an increased risk of second primary malignancies [14], where the latter are either iatrogenic or unrelated to treatment of the primary cancer. The type of iatrogenic secondary cancer the cancer survivor is at risk of depends on the initial cancer type and the type of treatment received, but hematological malignancies are among the most common type of secondary cancers [49]. While screening for new malignancies in cancer survivors should generally follow that in the general population, more intensive screening strategies may be required after certain treatments that increase the risk of certain cancer or in patients with hereditary cancer predisposition syndromes [14]. With certain hereditary syndromes, there is also the possibility that cancer survivors may require prophylactic surgeries depending on the evolution of the cancer (e.g., prophylactic salpingo-oophorectomy after a diagnosis of *BRCA1/2* mutated breast cancer) [50]. In such cases, it is also important that other members of the family receive genetic counseling [50].

### Cancer prevention and promotion of overall health and well-being

Lifestyle factors, including obesity, smoking, and alcohol intake, may place cancer survivors at increased risk of recurrence and/or developing subsequent primary cancers [51–54], and are also associated with an increased risk of comorbidities, cancer-related side effects, deterioration of QoL, and overall mortality [14, 55]. Indeed, there is evidence that reducing body weight and alcohol intake and achieving smoking cessation after a cancer diagnosis reduces the risk of cancer recurrence [56–58]. Also, environmental factors, such as air pollution, have been associated with the development of certain cancers [59]. Thus, promotion of overall health and well-being in cancer survivors is critical for future cancer prevention, overall health improvement, and improved QoL [14, 55, 60]. Despite a large body of evidence demonstrating beneficial effects of physical activity and exercise for cancer patients (e.g., increased physical fitness, improved health-related QoL, improvement in cancer-related fatigue, reduced likelihood of complications, reduced cancer mortality and recurrence) many cancer survivors report reduced physical activity after diagnosis, with cancer morbidity and fatigue often-cited barriers [61, 62]. Moreover, rapid rises in obesity have been reported among adult cancer survivors [55], underlining the importance of continued physical activity in this population.

## How to measure the needs of cancer survivors

To improve care of cancer survivors, the effects of cancer diagnosis and its treatment on patient QoL should be measured. Indeed, NCCN Guidelines® for survivorship recommend that the effects of cancer and treatment should be assessed at least annually in all cancer survivors [15]. To facilitate this, NCCN provides a sample survivorship assessment survey that can be used to monitor for the presence of common effects and evaluate their impact on QoL [15]. Also, the European Organisation for Research and Treatment of Cancer Quality of Life Group (EORTC QLG) has recently developed a cancer survivorship questionnaire to comprehensively capture the full range of physical and psychosocial issues that may affect QoL (QLQ-SURV111) [63]. This questionnaire has been pretested in a global phase 3 trial, and will be validated further in an ongoing phase 4 study.

A real-world study in Spain demonstrated that breast cancer patients surviving 5–10 years had an average of 26.5 healthcare service visits per year, mostly primary care [64], presenting multiple opportunities to monitor patients for effects. For example, it has been suggested that measuring lifestyle factors in a home setting could help to increase the feasibility of monitoring [65]. Use of sensors and wireless tools lowers patient burden, and facilitates longitudinal collection of potentially modifiable lifestyle factors such as dietary intake, body composition, alcohol consumption, smoking habits, and physical activity [65]. Also, cancer survivors have expressed a preference for internet delivery of healthy living interventions rather than telephone, particularly those further along the survivorship trajectory [60].

Further research is needed to explore optimal strategies for monitoring effects among cancer survivors (e.g., frequency, modality), and identify factors that may predict a higher risk of developing these sequelae. Given the importance of measuring and evaluating these effects, appropriate frameworks and protocols need to be established incorporating QoL scales, etc. Subsequent initiatives will be necessary to establish relevant recommendations.

## Establishing a care plan for cancer survivors

### Monitoring recommendations for cancer survivors

The ongoing care of cancer survivors should be an individualized, multidisciplinary, and coordinated process that aims to monitor for cancer recurrence, manage the effects of cancer and its treatment, and optimize the general health and wellbeing of people living with cancer [1, 14, 15, 17]. Firstly, reviewing a person’s cancer history, prior treatments, family history of cancer, and general health and lifestyle factors is important to assess their risk of cancer recurrence and of potential adverse events. Also, healthcare professionals should monitor and promote weight management, healthy diet and exercise, smoking cessation, and reduced alcohol consumption in these patients. Patients prefer a shared-care model for their cancer follow-up care and ongoing screening, where oncologists handle cancer follow-up care, and primary care providers (PCPs) manage general preventative healthcare and comorbidity issues [66].

### Physical needs and chronic medical conditions

Several review articles, consensus publications, and clinical practice guidelines provide recommendations for addressing the physiological needs of cancer survivors; these are outlined below for selected effects and summarized in Table 1.

Pain should be assessed at each follow-up visit; if the patient is experiencing new or acute pain, they should be assessed for cancer recurrence or late effects associated with specific therapies [15, 22]. Pain management should be discussed between physicians and patients to set realistic treatment goals, and should include a combination of pharmacological (e.g., non-opioid adjuvant analgesics, opioids in carefully selected patients) and non-pharmacological (e.g., exercise, use of heat and cold, massage, physiotherapy) strategies.

Recently published guidelines from ESMO provide evidence-based recommendations for the management of insomnia in cancer survivors [34]. ESMO recommends a first-line approach that addresses underlying conditions (e.g., hot flashes, pain, nocturia, maladaptive sleep behaviors, dysfunctional beliefs) combined with non-pharmacological therapies, with cognitive behavioral therapy for insomnia (CBT-I) recommended as the standard of care [34]. In the event that pharmacotherapy is prescribed for insomnia, the potential for drug–drug interactions should be borne in mind since many anticancer agents share similar metabolic pathways with psychotropic medications [34]. Evidence regarding the efficacy of exercise or bright light therapy for the treatment of insomnia in cancer survivors is currently limited [34].

### Psychological needs

NCCN Guidelines recommend that the psychological needs of cancer survivors be managed through regular screening for mental health issues such as fear of recurrence, anxiety, depression, trauma, and distress as part of their routine care [15]. Screening can be performed using the NCCN stress thermometer and problem list [67], or with validated measures such as the Patient Health Questionnaire (PHQ-9) for depression and the General Anxiety Disorder (GAD-7) questionnaire for anxiety [15]. To address the needs of survivors experiencing distress, patients may be referred to a mental health specialist or prescribed non-pharmacological interventions (e.g., cognitive behavioral therapy) and/or pharmacological treatments, depending on the diagnosis, acuteness and intensity of symptoms, safety of the survivor, and others factors [15].

### Social, employment, and financial needs

While a proportion of cancer survivors are not able to return to employment, work can have important psychosocial benefits, providing a sense of purpose, social connection, financial stability, and distraction from cancer-related worries [17]. Thus, it is important that physicians discuss cancer survivors’ concerns and desire to return to work. Discussion regarding negative effects of the disease and its treatment and the ability to work should be encouraged between survivors and their employers [17]. Occupational therapists also provide a useful role in helping to identifying workplace options and modifications that would enable the cancer survivor to continue in their previous work role [17]. Given the potential compliance implications, physicians should be prepared to discuss the financial effects of cancer treatment on patients; referral to financial support services may be appropriate [17].

In recent years, several countries in Europe have introduced legislation that protects the “right to be forgotten” and removes the legal requirement for cancer survivors to share their medical information with banking, financial, insurance, or employment services [68, 69]. These laws aim to protect cancer survivors from ongoing financial discrimination and are an important step towards reducing the economic burden associated with survivorship.

### Surveillance for recurrence and new cancers

Screening for new malignancies in cancer survivors is similar to that in the general population, and must balance the likelihood of developing cancer and risks associated with screening methods [14]. In this respect, individual patient factors (e.g., receipt of treatments that increase the risk of cancer, hereditary syndromes predisposing to cancer) must be considered [14]. However, further work is necessary to understand the most appropriate assessments and tests and their frequency of use in this patient population [14]. Monitoring for cancer recurrence, including the optimal type and frequency of surveillance tests, should be informed by cancer-specific clinical practice guidelines, such as those from the NCCN [70], ESMO [71], American Society of Clinical Oncology (ASCO) [72], and the National Institute for Health and Care Excellence (NICE) [73].

### Cancer prevention and promotion of overall health and well-being needs

Lifestyle factors (e.g., obesity, smoking, alcohol intake) of cancer survivors should be regularly monitored by physicians and appropriate measures taken to help ensure optimized health and well-being of the patient [65, 74]. Physicians should discuss the importance of weight management; promote healthy eating and exercise, smoking cessation, and limited alcohol intake; and, if necessary, refer patients to supportive services (e.g., weight-loss programs, dietary counselling, smoking cessation programs) [55, 65, 74, 75].

### Caring for special survivor populations

Survivors of pediatric malignancies are particularly vulnerable to the effects of cancer and its treatment [76]; therefore, the Children’s Oncology Group has developed specific guidelines for the follow-up of this population [77, 78]. These guidelines advocate for collaboration between primary care physicians, pediatric oncologists, patients, and families to develop and implement individualized survivorship care plans to address the needs of these patients; and highlight the importance of planning and systematic, ongoing follow-up as survivors transition from pediatric to adult healthcare systems.

Also, recent estimates suggest that approximately two-thirds of all cancer survivors in the US and Europe are aged > 65 years, and this proportion is predicted to grow as cancer prognoses improve and these populations age [3, 79, 80]. Older cancer survivors have complex care needs, largely due to the presence of comorbidities that can be exacerbated by cancer treatment, or increase the susceptibility of survivors to effects of cancer diagnosis [81]. The care of older cancer survivors may be optimized by geriatric assessments, which are multidimensional evaluations of functional, cognitive, psychosocial, and medical factors that can impact treatment decisions and goals, but which are not routinely captured in standard oncology assessments [82, 83]. Geriatric assessments are recommended in clinical practice guidelines for older patients receiving treatment for cancer [84, 85], and are increasingly being integrated into survivorship care plans and used to manage the ongoing health of those post-treatment [82].

### Integrated and multidisciplinary models of cancer survivorship care

At present, acute survivorship care in Spain typically follows a specialist-led model, whereby follow-up consultations are initially facilitated by oncologists and are primarily focused on monitoring for disease recurrence, progression, or subsequent primary cancers [14, 86]. However, specialist-led care often fails to address the full spectrum of effects and needs associated with cancer and its treatment, and will become increasingly unsustainable as the population of cancer survivors continues to grow around the world. As patients progress to the transitional, extended, and permanent phases of cancer survivorship, specialist-led follow-up decreases as patients move back into primary care (Fig. 1); this can be associated with feelings of worry and perceived loss of support [14, 16].

Several studies have evaluated alternative models of cancer survivorship care, including follow-up led by PCPs, shared between oncologists and general practitioners, or facilitated by specialist cancer nurses; overall, these approaches are at least as effective as the traditional specialist-led model of care [86]. In particular, the shared-care model may balance the management of cancer recurrence by oncologists with the management of secondary effects by general practitioners (Fig. 1), is associated with high patient satisfaction, and may incur lower healthcare costs than specialist-led care [86]. We advocate for the implementation of a multidisciplinary shared care model in future clinical practice, which could include the different specialties that can support the needs of cancer survivors, adapted to the specific and individual needs of each patient (e.g., psychology, nutrition, social work, cardiology, physiotherapy, etc.). However, we emphasize the importance of education and collaboration between specialists and PCPs, and recognize the challenge of applying a single model of care to a heterogeneous group of survivors and healthcare settings.

### Strategies to improve future cancer survivorship

Although great progress has been made in the diagnosis and treatment of cancer, leading to the increased prevalence of cancer survivors around the world, survivorship research has traditionally been overlooked. To address this imbalance, several groups have called for cancer survivorship research to be prioritized in international medical research agendas, with the aim of better understanding the current unmet needs of cancer survivors and developing evidence-based strategies to improve their health and QoL [87, 88].

Similarly, SEOM proposes a number of recommendations and future research areas that aim to address the care needs of cancer survivors in Spain. These include: a better understanding of the number of cancer survivors in Spain and the population that could benefit from this care; improved understanding of the current unmet needs of cancer survivors in Spain; raising awareness of the importance of cancer survivorship care; and improvement and implementation of multidisciplinary models of cancer survivorship care, with individualization of multidisciplinary survivorship care by pathology, by received treatment, and by patient. Additionally, SEOM recommends the implementation of national care strategies to correct disparities in the Spanish healthcare system, and encourage training of healthcare professionals who care for cancer survivors. Finally, SEOM also recommends enhanced patient participation and education in healthcare, and encourages future research and innovation. By elucidating the true burden of cancer survivorship on patients and society at large, implementing multidisciplinary models of care, and promoting future research and innovation, we endeavor to improve the lives of those living with, through, and beyond cancer.

## Conclusions

Cancer survivors, including those who have completed or are receiving treatment following a cancer diagnosis, frequently have physical, psychological, social, occupational, and financial care needs that can negatively impact health and QoL. The effects of cancer and its treatment, in addition to the general health and wellbeing of cancer survivors, are not adequately assessed and managed in current models of specialist-led care. We believe that the needs of cancer survivors could be better met by providing multidisciplinary care that involves primary care physicians, specialists, survivors, and their caregivers, and by future research that prioritizes this growing population.

## Data Availability

Data sharing is not applicable to this article as no new data were created or analyzed in this study.
